# From inflammation to inheritance: rethinking myocarditis as the first signal of desmosomal cardiomyopathy

**DOI:** 10.1186/s43044-026-00764-1

**Published:** 2026-07-06

**Authors:** Antoine Fakhry AbdelMassih, Anna Carinina Sankari, George Mallouka, Haya Sameh Ahmad Hattab, Jumana Abutaleb, Meera Albalooshi, Reem Abukhater, Shirin Khan

**Affiliations:** 1 Cairo University, Giza, Egypt; 2 Shaikh Khalifa Medical City, Abu Dhabi, United Arab Emirates; 3 University of Debrecen, Debrecen, Hungary; 4 Shaikh Khalifa Medical City, Abu Dhabi, United Arab Emirates; 5 Acıbadem University Atakent Hospital, Istanbul, Turkey

**Keywords:** Acute myocarditis, Desmosomal cardiomyopathy, Arrhythmogenic cardiomyopathy, Hot phase, Recurrent myocarditis, Genetic cardiomyopathy, Anti-DSG2 autoantibodies, Sudden cardiac death

## Abstract

**Background:**

Acute myocarditis has traditionally been regarded as an acquired inflammatory disorder of the myocardium, most commonly triggered by viral infection or immune-mediated injury. However, emerging evidence suggests that in a substantial subset of patients, myocarditis may represent the initial clinical manifestation of an underlying genetic cardiomyopathy rather than a purely inflammatory disease. Recent advances in molecular genetics, cardiac magnetic resonance (CMR) imaging, and translational pathology have highlighted a growing overlap between myocarditis and inherited cardiomyopathies, particularly those associated with desmosomal dysfunction.

**Main body:**

Variants in desmosomal genes—most commonly involving desmoplakin (DSP), plakophilin-2 (PKP2), and desmoglein-2 (DSG2)—have increasingly been reported in patients presenting with myocarditis-like syndromes characterized by chest pain, troponin elevation, and CMR findings fulfilling the Lake Louise criteria. Observational and registry studies suggest that some of these patients subsequently experience recurrent episodes of myocardial injury, ventricular arrhythmias, and progressive fibrotic or fibrofatty myocardial remodeling, eventually developing phenotypic features consistent with arrhythmogenic cardiomyopathy (ACM). ACM is used throughout this review as an inclusive umbrella term encompassing classical arrhythmogenic right ventricular cardiomyopathy (ARVC) as well as biventricular and left-dominant variants. Experimental and translational studies further suggest mechanistic links between desmosomal dysfunction and myocardial inflammation, including DAMP-mediated innate immune activation and the generation of anti-desmoglein-2 (anti-DSG2) autoantibodies. While these observations support a possible role for autoimmunity, the causal contribution of these autoantibodies to myocardial injury and disease progression remains incompletely established and constitutes a proposed rather than confirmed model.

**Conclusion:**

The emerging relationship between myocarditis and desmosomal cardiomyopathy challenges the traditional distinction between inflammatory and genetic myocardial disease. Integrating genetic testing, advanced CMR imaging, arrhythmic surveillance, and emerging immunological biomarkers may facilitate earlier recognition of genetically mediated cardiomyopathy presenting as myocarditis-like episodes. Further prospective and mechanistic studies are needed to establish causality and evaluate therapeutic implications.

## Background

Acute myocarditis has traditionally been regarded as an acquired inflammatory disease of the myocardium, most commonly triggered by viral infection or immune-mediated mechanisms, with outcomes ranging from complete recovery to chronic dilated cardiomyopathy [1, 2]. However, the classical dichotomy between “inflammatory” and “genetic” myocardial disease is increasingly difficult to sustain. This difficulty is not merely one of classification but reflects shared pathophysiological mechanisms: structural vulnerability arising from genetic defects in proteins critical to cardiomyocyte integrity may lower the threshold for inflammatory activation, while concurrent or preceding inflammation may accelerate the phenotypic expression of an otherwise subclinical inherited substrate [3, 4]. Importantly, much of the supporting evidence derives from observational studies, tertiary-centre registries, and case series, and a clear distinction must be maintained throughout this review between what is established, what is probable, and what remains mechanistically plausible but unproven.

Advances in cardiac imaging, molecular pathology, and genetic testing have progressively revealed an overlap between myocarditis and inherited cardiomyopathies, particularly arrhythmogenic and dilated phenotypes [5, 6]. This overlap can manifest as myocarditis-like presentations—characterized by chest pain, troponin elevation, electrocardiographic changes, and CMR features fulfilling Lake Louise criteria—that are followed by recurrent inflammatory episodes, ventricular arrhythmias, or progressive myocardial dysfunction disproportionate to the apparent inflammatory burden [7, 8]. These observations have prompted a reconsideration of myocarditis as, in a subset of patients, the initial clinical expression of a genetically determined myocardial disease [9].

The genetic substrate most frequently implicated involves desmosomal genes, variants in which are the molecular basis of arrhythmogenic cardiomyopathy (ACM)—used throughout this manuscript as the preferred inclusive umbrella term, encompassing the originally described right-dominant form (historically termed arrhythmogenic right ventricular cardiomyopathy, ARVC) as well as biventricular and left-dominant variants [10, 11]. This distinction is clinically relevant: left-dominant ACM, particularly that associated with *DSP* variants, characteristically presents with subepicardial to mid-myocardial fibrosis and left ventricular inflammation, a CMR pattern that closely resembles isolated myocarditis [12].

Regarding prevalence, estimates of pathogenic variant yield in myocarditis cohorts vary substantially by case-mix. A systematic review and meta-analysis reported pooled prevalence figures of 21.9% (95% CI 14.3%–30.5%) in complicated adult myocarditis and 44.5% (95% CI 22.7%–67.4%) in children, with desmosomal variants predominating in uncomplicated cases (64% of variant-positive cases) and sarcomeric variants more prevalent in complicated adult disease (58%) [13]. In contrast, the population-based cohort study by Lota and colleagues—336 consecutive unselected myocarditis patients—identified DCM- or ACM-associated variants in 8% of cases versus less than 1% of healthy controls (P = 0.0097), driven predominantly by *DSP* truncating variants in patients with preserved ejection fraction and ventricular arrhythmia [14]. The contrast between the 8% yield in unselected cohorts and the 22–45% yield in referral populations reflects case-mix differences and confirms that published figures from specialist centres overestimate variant prevalence in unselected myocarditis. These data nonetheless establish that genetic cardiomyopathy—particularly ACM—can present with, and may be unmasked by, inflammatory episodes.

This article is accordingly framed as a focused narrative review aimed at synthesizing the available clinical, genetic, imaging, and translational evidence bearing on the hypothesis that myocarditis-like presentations may constitute an early or recurrent inflammatory manifestation of desmosomal cardiomyopathy. It appraises the mechanistic frameworks proposed to explain this overlap, discusses alternative hypotheses and conflicting evidence, and translates these insights into a clinically applicable framework for everyday cardiology practice.

## Methods

### Review design

This article is a focused narrative review conducted according to the principles recommended for expert synthesis reviews in cardiovascular medicine. The review was not registered and did not employ a systematic or scoping methodology; accordingly, it does not claim to provide an exhaustive or unbiased census of literature. Rather, it aims to synthesize available evidence and appraise mechanistic frameworks, with explicit characterization of evidence quality throughout.

### Literature search strategy

A targeted literature search was performed in PubMed/MEDLINE from database inception through March 2025, using combinations of Medical Subject Headings and free-text terms including: “myocarditis,” “recurrent myocarditis,” “arrhythmogenic cardiomyopathy,” “desmosomal cardiomyopathy,” “desmoplakin,” “plakophilin-2,” “desmoglein-2,” “desmocollin-2,” “hot phase,” “inflammatory cardiomyopathy,” “genetic cardiomyopathy,” “anti-DSG2 autoantibodies,” “intercalated disc,” and “late gadolinium enhancement.” Search terms were combined using Boolean operators and were not restricted by language. Reference lists of retrieved articles were manually screened for additional sources, and seminal papers identified through expert knowledge were included at author discretion.

### Inclusion and exclusion criteria

Included sources comprised original research articles (case reports, observational cohort studies, registry analyses, and genetic screening studies), mechanistic and translational studies examining desmosomal biology in cardiac tissue, and review articles or consensus documents directly relevant to the pathophysiology or clinical management of desmosomal cardiomyopathy and myocarditis. No study design hierarchy was applied as a formal inclusion criterion, given the scarcity of large randomized trials, but the level of evidence supporting each statement is indicated in the text. Excluded were abstracts without full-text publication, studies focused exclusively on non-cardiac desmosomal disease without direct relevance to cardiac pathophysiology, and articles examining cardiomyopathies unrelated to the desmosomal-inflammatory interface. Studies of purely dermatological desmosomal biology were included only where they provided mechanistic analogies directly applicable to cardiomyocyte junction biology and are identified as such in the text.

### Evidence synthesis

Given the observational and largely hypothesis-generating nature of available evidence, formal meta-analytic synthesis was not performed. Evidence is presented narratively with explicit characterization of study design, sample size, and key limitations. Statements derived from single case reports or small series are identified as such. Mechanistic propositions not validated in human cardiac tissue are described as hypothetical or proposed rather than established, and conflicting evidence is discussed alongside the primary interpretation.

## Discussion

### Myocarditis-like episodes in desmosomal cardiomyopathies

A subset of patients presenting with acute myocarditis may harbour an underlying genetic cardiomyopathy, most commonly involving pathogenic variants in desmosomal proteins. ACM encompasses a group of inherited myocardial disorders characterized by ventricular arrhythmias, myocardial fibrosis, and progressive ventricular dysfunction, in which inflammatory myocardial injury—manifesting as acute myocarditis-like episodes—is now recognized as an important and potentially early component of the disease process [15, 16].

Desmosomes are intercellular junctions within the cardiomyocyte intercalated disc that provide mechanical coupling between cells. In the adult mammalian heart, these junctions exist not as classical desmosomes but as a cardiac-specific hybrid structure termed the area composita, in which desmosomal proteins (including DSG2, DSC2, plakophilin-2, and desmoplakin) co-localize with adherens junction components (N-cadherin, α-catenin, β-catenin) within the same electron-dense plaque [17]. Pathogenic variants in desmosomal genes do not therefore disrupt an isolated desmosome—they destabilize the entire area composita complex, impair mechanical coupling, and disrupt molecular cross-talk between its constituent proteins. Consequent cardiomyocyte vulnerability to mechanical stress may trigger cell death and secondary inflammatory responses that mimic acute myocarditis clinically [18, 19].

The multicentre retrospective study by Ammirati and colleagues (97 patients, 23 hospitals) remains the largest dataset examining this association in a clinically ascertained myocarditis population. *DSP* variants accounted for the largest proportion of identified mutations. Patients typically presented with chest pain and troponin elevation with preserved systolic function, and those carrying desmosomal variants had significantly higher rates of recurrent inflammatory episodes, ventricular arrhythmias, and heart failure during follow-up compared with gene-negative counterparts [20]. These findings are consistent with the interpretation that, in genotype-positive patients, inflammatory episodes represent recurrent disease activity within an ACM substrate, though the retrospective referral-centre design limits causal inference.

Recurrent inflammatory episodes have emerged as a clinical feature that should prompt consideration of an underlying genetic cardiomyopathy. In a longitudinal cohort of patients with recurrent apparent myocarditis, a proportion subsequently developed phenotypic features of ACM over follow-up, with genetic testing identifying pathogenic variants in desmosomal genes—most commonly *DSP* and *PKP2*—in a number of cases [21]. These inflammatory episodes often preceded structural manifestations by several years, supporting the concept that recurrent myocarditis-like presentations may represent an early, pre-structural phase of ACM [22]. The evidence base for this trajectory, however, remains limited to relatively small observational cohorts, and the proportion of patients with recurrent myocarditis who ultimately develop overt ACM has not been precisely quantified.

Among desmosomal genes, *DSP* variants have been particularly associated with inflammatory myocardial injury. Poller and colleagues described two brothers with truncating *DSP* variants who experienced repeated episodes of chest pain, troponin elevation, electrocardiographic changes, and multifocal subepicardial late gadolinium enhancement (LGE) on CMR without identifiable infectious triggers—a pattern consistent with mechanically driven inflammatory injury rather than viral myocarditis [23]. This familial observation illustrates how DSP-related disease can present recurrently with an apparently inflammatory phenotype, reinforcing the importance of genetic evaluation in this context. Importantly, the paediatric literature deserves separate consideration: in children and adolescents, myocarditis-like episodes are often the only initial manifestation of ACM and may be mistaken for fulminant viral myocarditis, while in adults they more frequently overlap with an already fibrotic substrate [24].

In a pilot study of 25 consecutive symptomatic probands with pathogenic cardiomyopathy gene variants, Peretto and colleagues found that the majority carried desmosomal mutations—most commonly *DSP*—and presented with myocarditis-like episodes marked by chest pain and ventricular arrhythmias, with extensive replacement fibrosis and limited necrosis on biopsy [25]. These findings suggest inflammation may act as a disease modifier rather than the primary driver of injury, though interpretation of biopsy findings is complicated by the substantial false-negative rate of conventional endomyocardial biopsy (EMB) in this context, as discussed below.

### Area composita dysfunction, innate immune activation, and the proposed autoimmune cascade (Fig. 1 summarizes the proposed hypothesis)

**Fig. 1:**
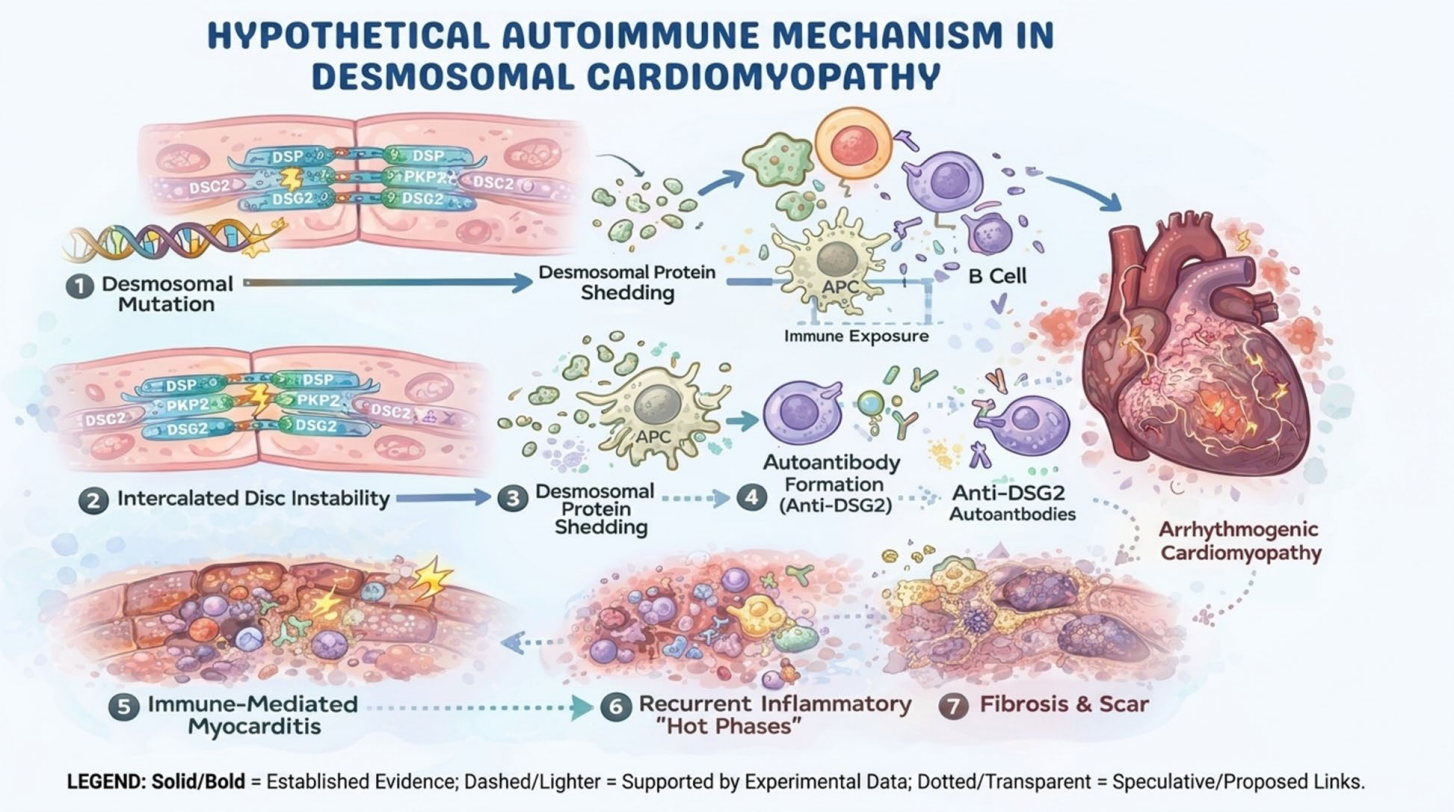
This figure illustrates the key stages of the experimental process, highlighting the methodology used to measure the variables of interest. Each step is depicted with detailed annotations to clarify the techniques applied and the significance of each phase in relation to the overall hypothesis

One proposed mechanism linking pathogenic variants in desmosomal genes to inflammatory myocardial injury involves a cascade of structural, innate immune, and potentially adaptive immune events initiated at the level of the intercalated disc. It is important to state at the outset that the complete causal sequence described below constitutes a working model—not established human pathophysiology—and should be interpreted accordingly.

#### Cell death, DAMP release, and innate immune activation

Mechanical disruption of the area composita complex renders cardiomyocytes vulnerable to injury under conditions of increased haemodynamic load, including exercise. Multiple modes of cardiomyocyte death appear to operate in parallel. Apoptosis has been documented histologically in heterozygous *DSP* mutant mouse models, where patchy left ventricular fibrosis and apoptotic cardiomyocytes were identified in the absence of significant systolic dysfunction [26]. Cardiomyocyte necrosis—including calcifying necrosis—is a prominent feature of *DSG2* ablation models and is the likely primary driver of acute DAMP release [27]. More recently, necroptosis has been proposed as an additional regulated cell death mechanism that amplifies DAMP release relative to apoptosis, though direct evidence for necroptotic activation in human desmosomal cardiomyopathy tissue is currently limited [28].

Released DAMPs—including cytoplasmic nucleic acids, heat shock proteins, and HMGB1—activate pattern recognition receptors on resident macrophages within the myocardial interstitium, Resident cardiac macrophages, derived from the yolk sac lineage and present in substantial numbers in the non-inflamed myocardium, are likely the first innate immune responders to desmosomal protein shedding and cell death [29]. Their activation through Toll-like receptors (TLRs) and NLRP3 inflammasome signaling drives NF-κB-mediated cytokine production—including interleukin-1β, interleukin-6, and tumour necrosis factor-α—establishing the pro-inflammatory microenvironment that recruits circulating monocytes and lymphocytes to the site of injury [30]. The lymphocytic infiltrates observed histologically in myocardial specimens from ACM patients are consistent with this innate immune activation, though lymphocytic myocarditis is a non-specific finding and does not independently confirm an autoimmune pathogenesis [31].

#### Connexin-43 mislocalisation and arrhythmogenesis

Area composita disruption has direct electrophysiological consequences independent of active inflammation. Multiple lines of experimental evidence demonstrate that desmosomal protein variants promote redistribution of connexin-43 (Cx43)—the principal ventricular gap junction protein—away from the intercalated disc to lateral sarcolemmal membrane and cytoplasmic compartments [32, 33]. In heterozygous DspS311A mice, Cx43 mislocalisation was detectable from one month of age, preceding structural remodeling, indicating that it reflects a primary junctional coupling defect rather than a secondary consequence of fibrosis [34]. Lateralized Cx43 creates conduction heterogeneity and anisotropy within the ventricular wall, constituting an arrhythmogenic substrate independent of the inflammatory state—suggesting that arrhythmic risk in ACM may be present even before fibrosis is detectable by CMR [35].

#### Anti-DSG2 autoantibodies: evidence, controversies, and clinical status

The potential adaptive immune component of this cascade involves the generation of autoantibodies directed against desmosomal proteins exposed by area composita disruption. Antibodies targeting DSG2 have received the most attention. In the original investigation by Chatterjee and colleagues (2018), anti-DSG2 antibodies were detected in all 12 and 25 of 25 subjects with definite ARVC across two independent cohorts, and in 7 of 8 borderline subjects, while absent in healthy controls and patients with hypertrophic or dilated cardiomyopathy. Antibody levels correlated with premature ventricular contraction burden (r = 0.70), and antibodies impaired Cx43-mediated gap junction function in vitro [36].

These findings require important qualification, however. First, the original study did not include patients with myocarditis in its control population—a critical omission when evaluating specificity for ACM against a clinically relevant comparator. Second, the assay methodology has not been standardized, and the correlation between ELISA-based anti-DSG2 detection and the validated immunofluorescence-based technique for anti-intercalated disc autoantibodies (AIDAs) has not been established [37]. Third, the 2024 multicentre study by Giordani and colleagues (n = 77 ARVC, n = 91 myocarditis/DCM, n = 27 immune-mediated disease, n = 50 controls)—which for the first time included myocarditis patients as a comparator group—reported an ELISA sensitivity of 72.3% with 100% specificity at an optimal cut-off, yielding an area under the curve of 0.823 [38]. This represents a substantially lower sensitivity than the original reports. Furthermore, anti-DSG2 positivity was detected in a proportion of patients with cardiac sarcoidosis [39], and in post-COVID-19 serum samples [40], raising the possibility that the antibody may reflect a shared response to intercalated disc injury of any cause rather than being specific to ACM aetiology.

The fundamental question of whether anti-DSG2 antibodies are causal, contributory, or epiphenomenal therefore remains unresolved. Three positions are possible. In the causal model, anti-DSG2 IgG binds to cardiomyocyte surface DSG2 and directly activates inflammatory signaling—potentially via Fc receptor-mediated immune cell recruitment, complement classical pathway activation, or antibody-dependent cellular cytotoxicity (ADCC) mediated by natural killer cells and macrophages. In the contributory model, anti-DSG2 antibodies amplify an inflammatory process initiated by mechanical injury without being sufficient to cause disease independently. In the epiphenomenal model, these antibodies arise as a consequence of cardiomyocyte death and reflect the extent of myocardial damage without contributing to its pathogenesis—analogous to anti-myosin antibodies in viral myocarditis [41]. Available human data are insufficient to distinguish between these models definitively. Anti-DSG2 testing is currently a research tool and is not available for routine clinical use; its ability to differentiate hot-phase ACM from acute viral myocarditis in real time has not been prospectively evaluated. [42]

### Alternative hypotheses for myocarditis-like episodes in genotype-positive patients: a critical appraisal

The identification of a pathogenic or likely pathogenic variant in a cardiomyopathy-associated gene in a patient presenting with myocarditis does not establish that the genetic variant is the cause—or even a contributing factor—of the current episode. At least five mechanistically distinct hypotheses can account for the co-occurrence of a genetic variant and a myocarditis-like presentation. These are not mutually exclusive; in individual patients, more than one may operate simultaneously or sequentially, and the dominant hypothesis may shift as the disease course evolves.

#### Hypothesis 1

Two-Hit Model (Viral Infection Superimposed on Structural Vulnerability)

The most clinically prevalent and biologically plausible alternative is that genotype-positive patients develop genuine viral myocarditis—triggered by a cardiotropic pathogen such as parvovirus B19, adenovirus, or enterovirus—on the same mechanistic basis as genotype-negative patients, but with the structural vulnerability of the area composita amplifying the degree of inflammatory injury [43, 44]. The genetic variant in this model functions as a necessary predisposing condition that lowers the threshold for overt myocardial damage when infection occurs, rather than as a sufficient cause of inflammation in its own right. Cardiotropic viruses have been detected in myocardial specimens from a subset of ACM patients, and Lota and colleagues explicitly proposed that genotype-positive individuals may remain phenotypically silent until an environmental trigger—including viral myocarditis—precipitates phenotypic expression [14].

#### Hypothesis 2

 Exercise-Triggered Sterile Inflammation (Mechanical Failure Model)

A second mechanistic pathway involves high-intensity exercise as the proximate trigger for sterile myocardial inflammation through purely mechanical disruption of the area composita, in the complete absence of infection or adaptive immune activation [23, 45]. This hypothesis is most strongly supported by the observations of Poller and colleagues, where recurrent episodes of chest pain, troponin elevation, and subepicardial LGE occurred consistently in association with physical exertion, with exhaustive infectious workup uniformly negative. It is further supported by PKP2 mouse models demonstrating that high-endurance exercise accelerates area composita remodeling, promotes fibrosis, and increases arrhythmic burden [46]. Clinically, the sterile inflammation model implies that exercise restriction is not merely supportive management but a direct disease-modifying intervention—a distinction with profound implications for long-term physical activity guidance that differs fundamentally from the temporary restriction recommended after viral myocarditis.

#### Hypothesis 3

Primary Inflammatory Myocarditis with Incidental Genetic Findings

A scientifically important alternative is that a genotype-positive patient presenting with myocarditis is experiencing genuine primary inflammatory myocarditis—viral, immune-mediated, or idiopathic—in which the genetic variant is an incidental co-occurrence unrelated to the current episode. This hypothesis is quantitatively grounded: approximately 0.4% of the healthy control population carry ACM-associated truncating variants [14], meaning that a non-trivial proportion of patients with viral myocarditis will by chance carry a pathogenic variant that exerts no causal role in the presenting episode. This hypothesis is most credible when the episode is a first presentation with an identifiable viral trigger, when the CMR pattern is consistent with typical viral disease, and when complete LGE regression occurs on follow-up CMR at 3–6 months [47]. Autoantibodies detected in such patients may reflect non-specific tissue damage from any cause rather than a disease-specific autoimmune process.

#### Hypothesis 4

Cardiac Sarcoidosis and Other Phenocopies

Cardiac sarcoidosis (CS) deserves explicit consideration as a phenocopy of ACM and myocarditis that is misclassified in a clinically significant proportion of cases. Vasaiwala and colleagues reported that 15% of initially diagnosed ACM cases were reclassified as CS based on invasive findings, and the 2010 Task Force criteria—including the Padua extension—lack sufficient specificity to reliably distinguish CS from ACM, particularly in the left-dominant or biventricular phenotype [48, 49]. CS can fulfil Task Force structural, depolarization, and repolarization criteria, produce biventricular LGE with subepicardial and mid-wall distribution, and present with recurrent ventricular arrhythmias and high-degree conduction disease—all features shared with genetic ACM during inflammatory episodes. Giant cell myocarditis (GCM), eosinophilic myocarditis, and drug-related hypersensitivity myocarditis are further phenocopies whose identification requires EMB and carries urgent management implications—immunosuppression is life-saving in GCM and must not be delayed pending genetic evaluation [50, 51].

#### Hypothesis 5

Inflammatory Myocarditis as an Epigenetic Modifier Unmasking Latent Genetic Disease

The fifth hypothesis positions acute myocarditis from any cause as a structural and potentially epigenetic catalyst that accelerates phenotypic expression of an otherwise latent genetic cardiomyopathy. Inflammation from viral or immune-mediated myocarditis may promote area composita remodeling, Cx43 mislocalisation, and fibrosis deposition in a genotype-positive individual, effectively shortening the latency period between genetic susceptibility and overt ACM [52]. This hypothesis is supported by case-level observations, including a report of biventricular ACM developing 29 years after an initial episode of myocarditis, with a *PKP2* pathogenic variant identified on subsequent genetic testing [53]. It argues strongly for long-term follow-up of all genotype-positive patients after a myocarditis episode, regardless of apparent clinical recovery.

### Non-desmosomal genetic substrates associated with myocarditis-like presentations

Although desmosomal variants have been the most extensively studied genetic substrate in inflammatory cardiomyopathy, a growing body of evidence indicates that pathogenic variants in several non-desmosomal genes can produce clinically indistinguishable myocarditis-like presentations (Table 1) [54, 55].

**Table 1:** Principal genetic substrates associated with myocarditis-like presentations: desmosomal and non-desmosomal genes

Gene	Protein	Inflammatory mechanism	Myocarditis-like phenotype	CMR pattern	Arrhythmia risk	TF criteria met?
*DSP*	Desmoplakin; area composita anchor	Mechanical uncoupling → necrosis → DAMPs; anti-DSG2 generation	Recurrent chest pain, troponin elevation; LV-dominant hot phase	Subepicardial LGE, LV ring pattern; T2 elevation	VT/VF; LV dysfunction; fibrofatty replacement	Often yes
*PKP2*	Plakophilin-2; desmosomal scaffold	RV mechanical stress; Hippo pathway; necrosis-driven inflammation	RV-dominant hot phase; classic ARVC phenotype	RV/biventricular LGE	VT; RV dilation	Often yes
*DSG2/DSC2*	Desmoglein-2/Desmocollin-2; cadherin adhesion	Antigenic exposure; complement activation; anti-DSG2 IgG	Myocarditis-like episodes; biventricular involvement	Biventricular LGE; subepicardial and mid-wall	VT; biventricular ACM	Sometimes
*FLNC*	Filamin C; cytoskeletal Z-disc protein	Z-disc disruption → DAMPs; NF-κB activation; autophagic stress	Recurrent myocarditis-like; LV-dominant; clinically resembles viral myocarditis	Subepicardial/epicardial LV fibrosis; ring-like or inferior-lateral; Lake Louise criteria frequently met	Malignant arrhythmia risk despite preserved LVEF	Usually not
*LMNA*	Lamin A/C; nuclear envelope integrity	Nuclear lamina disruption → DNA breaks → cGAS-STING → type I IFN; NF-κB	Inflammatory cardiomyopathy; conduction disease co-present	Mid-wall and subepicardial LGE; septal involvement	High-degree AV block; VT/VF; rapid DCM; high SCD risk	No
*PLN*	Phospholamban; SR calcium pump regulator	Calcium dysregulation → ER stress → inflammatory cytokines (proposed)	Recurrent myocarditis-like episodes (p.Arg14del)	Subepicardial to mid-wall; inferior-lateral LV	VT; sudden death; progressive LV dilation	No
*TTN*	Titin; sarcomeric elastic protein	Truncating variants impair mechanical signalling; stretch-activated inflammatory pathways	Myocarditis with reduced LVEF; TTNtv in 7% of unselected cohort (Lota et al.)	Mid-wall LGE; diffuse LV involvement	VT; progressive DCM	No
*RBM20*	RNA-binding motif 20; titin splicing regulator	Aberrant titin isoforms → mechanical vulnerability; inflammation under investigation	Inflammatory cardiomyopathy phenotype; myocarditis-like episodes in young patients	Biventricular LGE; mid-wall and subepicardial patterns	VT/VF; biventricular DCM; high SCD risk	No

*ACM*  arrhythmogenic cardiomyopathy, *AV*  atrioventricular, *CMR*  cardiac magnetic resonance, *DAMPs*  damage-associated molecular patterns, *DCM*  dilated cardiomyopathy, *ER*  endoplasmic reticulum, *IFN*  interferon, *LGE*  late gadolinium enhancement, *LV*  left ventricle, *LVEF*  LV ejection fraction, *NF-κB*  nuclear factor kappa-B, *RV*  right ventricle, *SCD*  sudden cardiac death, *SR*  sarcoplasmic reticulum, *TF*  Task Force, *VT*  ventricular tachycardia, *VF * ventricular fibrillation

TF criteria: 2010 Task Force criteria revised by Padua 2020 extension

*FLNC* truncating variants are among the most clinically relevant non-desmosomal causes of recurrent inflammatory episodes. Filamin C is a Z-disc structural protein whose disruption impairs cytoskeletal integrity and Mechano-sensing, provoking mechanical cardiomyocyte injury and DAMP-mediated NF-κB activation.—producing episodes of chest pain, troponin elevation, and CMR findings fulfilling Lake Louise criteria [56, 57]. The resulting fibrosis is characteristically subepicardial and epicardial, predominantly affecting the left ventricle—a distribution that closely resembles DSP-related disease and is readily misattributed to viral myocarditis. Importantly, *FLNC* truncation carriers frequently do not meet Task Force criteria for ACM at presentation, and the arrhythmic risk appears disproportionate to apparent structural disease [58].

*LMNA* variants engage a mechanistically distinct pathway. Lamin A/C is a nuclear envelope constituent whose disruption compromises nuclear structural integrity, predisposing cardiomyocytes to DNA strand breaks and cytoplasmic chromatin release. Cytoplasmic double-stranded DNA activates the cGAS-STING innate immune sensing pathway, triggering type I interferon production and NF-κB-mediated cytokine release—a mechanism more analogous to innate immune activation in viral myocarditis than to the autoimmune cascade proposed for desmosomal disease [59, 60]. The co-occurrence of high-degree atrioventricular block or progressive conduction disease alongside apparent myocarditis is a characteristic combination that should prompt targeted *LMNA* testing.

*PLN* variants—particularly the p.Arg14del founder variant—have been associated with recurrent myocarditis-like episodes in a subset of carriers, with a CMR pattern involving the subepicardial and mid-wall left ventricle in an inferior-lateral distribution. The proposed mechanism involves calcium dysregulation via impaired sarcoplasmic reticulum calcium handling, leading to endoplasmic reticulum stress and mitochondrial dysfunction that amplify DAMP-driven inflammatory signalling [61]. Human evidence for direct PLN-mediated inflammation remains limited, and inflammatory episodes in this context should currently be regarded as a proposed rather than established feature of the disease.

*TTN* truncating variants were identified in 7% of an unselected myocarditis cohort by Lota and colleagues, enriched specifically among patients presenting with reduced left ventricular ejection fraction (LVEF) [14]. The mechanistic link between titin truncation and inflammatory activation is less well characterized than for *FLNC* or *LMNA* disease, and *TTN* variants may in some cases represent susceptibility modifiers rather than primary causal substrates in isolation [62].

*RBM20* variants, affecting titin splicing, have also been reported in patients with inflammatory cardiomyopathy phenotypes. Biventricular LGE, atypical distribution, and a high risk of sudden cardiac death characterize this relatively recently recognized entity [63]. The mechanistic link to inflammation remains under investigation but may involve aberrant titin isoform production and secondary mechanical vulnerability.

Considered together, these non-desmosomal substrates reinforce the case for systematic genetic evaluation in patients presenting with recurrent or phenotypically atypical myocarditis. The subepicardial or epicardial LV fibrosis pattern on CMR—frequently invoked as distinguishing of DSP-related ACM—is shared by *FLNC* truncation carriers and variably by *PLN* and *LMNA* variants, limiting its discriminatory value at the individual patient level. The mechanistic heterogeneity across gene groups—autoimmune and antigen-driven in desmosomal disease, innate immune and DNA-sensing in *LMNA*, Mechan sensing-driven DAMP release in *FLNC*—has potential therapeutic relevance: if mechanism-targeted interventions are eventually developed, a gene-specific rather than pan-inflammatory approach may be required [64].

## Diagnostic and therapeutic framework for myocarditis-like episodes in genetic cardiomyopathy

The clinical convergence between myocarditis and genetic cardiomyopathy—particularly ACM and desmosomal variants—presents a diagnostic and therapeutic challenge that is not adequately addressed by standard myocarditis management algorithms. The framework described in this section integrates clinical evaluation, CMR imaging, genetic testing, arrhythmic risk stratification, and exercise restriction guidance into a structured approach for patients in whom a genetic substrate is suspected or confirmed (Table 2). The level of evidence supporting each recommendation is indicated throughout; in the absence of randomized trial data, recommendations reflect expert consensus and best available observational evidence.

**Table 2:** Diagnostic and therapeutic framework: viral myocarditis versus genetic hot-phase ACM across five clinical domains

Domain/feature	Detail	Viral/Immune myocarditis	Genetic hot-phase (ACM/DSP/FLNC)
Chest pain	Character	Pleuritic or positional; associated with pericarditis in ~ 30%	Sharp, exertional; often without pericarditis; may follow physical activity
Troponin	Pattern	Elevated; modest rise; correlates with LV dysfunction	May be disproportionately low relative to CMR fibrosis extent—key red flag
Systolic function	LVEF	Often mildly reduced; correlates with troponin	LVEF frequently preserved despite extensive LGE—key distinguishing feature
Arrhythmia	Type and severity	Atrial arrhythmias, VPBs; sustained VT uncommon	NSVT or VT frequent with preserved LVEF; exercise-provoked VT characteristic
Recurrence	Probability	Rare (< 5% in idiopathic viral myocarditis)	Common; hallmark trigger for genetic evaluation
LGE distribution	Pattern	Lateral/inferior LV; subepicardial or midwall; patchy	Ring-like LV (DSP); epicardial LV (FLNC); biventricular (PKP2/DSG2); inferior-lateral (PLN)
LGE extent	Relative to troponin	Proportionate to clinical severity	Often extensive; disproportionate to troponin elevation—key red flag
Serial CMR	Follow-up trajectory	LGE regresses; T2 normalises within 3–6 months	LGE stable or progressive; new areas may appear; serial imaging essential
Immunosuppression	Indication	Indicated in autoimmune (non-viral) myocarditis confirmed by EMB; not routine in viral disease	Not recommended outside trials; corticosteroids carry risk of worsening ACM; CS must be excluded first
Exercise restriction	Duration and criteria	Minimum 3 months; return after CMR resolution	Complete restriction ≥ 3–6 months; competitive sport contraindicated in genotype-positive patients; annual reassessment
ICD threshold	Indication	Standard: LVEF ≤ 35% persistent after 3–6 months; aborted SCA	Lower threshold in DSP/LMNA; ICD reasonable with extensive LGE + NSVT even with preserved LVEF
Family screening	Mandate	Not indicated unless familial clustering identified	Mandatory cascade genetic testing; clinical evaluation (CMR + ECG + Holter) in all gene-positive relatives annually
Surveillance	Schedule	CMR at 3–6 months; clinical review at 1 year; discharge if complete recovery and no arrhythmia	CMR annually; Holter + exercise test 6-monthly; lifelong specialist follow-up; consider ILR in high-risk patients

*ACM*  arrhythmogenic cardiomyopathy, *CMR*  cardiac magnetic resonance, *CS*  cardiac sarcoidosis, *DSP  *desmoplakin, *EMB*  endomyocardial biopsy, *FLNC*  filamin C, *ICD*  implantable cardioverter-defibrillator, *ILR*  implantable loop recorder, *LGE * late gadolinium enhancement, *LMNA*  lamin A/C, *LV*  left ventricle, *LVEF*  LV ejection fraction, *NSVT*  non-sustained ventricular tachycardia, *PKP2*  plakophilin-2, *PLN*  phospholamban, *SCA*  sudden cardiac arrest, *VPB*  ventricular premature beat, *VT*  ventricular tachycardia

### Clinical evaluation and indications for genetic testing

Patients presenting with a myocarditis-like episode—defined by the combination of chest pain, troponin elevation, and CMR or ECG changes consistent with myocarditis—should be evaluated systematically for features that raise the pre-test probability of an underlying genetic substrate. Table 2 provides a structured “When to Suspect Genetic Disease” framework.

High-probability features that should trigger early referral for comprehensive cardiomyopathy gene panel testing (minimum panel: *DSP*, *PKP2*, *DSG2*, *DSC2*, *FLNC*, *LMNA*, *PLN*, *TTN*, *SCN5A*, *RBM20*) include: (1) recurrence of inflammatory episodes (two or more documented events); (2) ventricular tachycardia or fibrillation disproportionate to apparent inflammatory burden; (3) preserved LVEF with significant ventricular arrhythmia; and (4) a family history of ACM or sudden cardiac death before the age of 50 years [65, 66]. Moderate-probability features providing supportive trigger include: subepicardial or ring-like LGE on CMR; biventricular LGE involvement; LGE extent disproportionate to the degree of troponin elevation; absence of pericardial involvement; and a CMR pattern atypical for isolated viral myocarditis [67, 68]. Genetic testing should be pursued independently of whether CMR formally meets Lake Louise criteria, since hot-phase ACM episodes frequently satisfy myocarditis imaging criteria while arising from a desmosomal substrate.

### Endomyocardial biopsy: indications and limitations

Endomyocardial biopsy (EMB) retains a role in selected patients and should be performed when GCM, CS, or eosinophilic myocarditis is suspected, or when the distinction between inflammatory and non-inflammatory cardiomyopathy has immediate therapeutic consequences [69]. Its utility for detecting myocardial inflammation in genetic cardiomyopathy is substantially limited by the distribution of disease. Standard right ventricular trans endocardial EMB achieves a sensitivity of approximately 42% for myocarditis in acute presentations using Dallas histological criteria, falling to 23% in subacute or chronic cases; even combined multi-site sampling achieves only 64% per-case sensitivity [70]. In ACM and related desmosomal disorders, inflammation is characteristically patchy, subepicardial, and mid-myocardial—regions anatomically inaccessible to standard biopsy. A negative EMB result in a patient with CMR evidence of subepicardial inflammation should therefore increase rather than decrease clinical suspicion for an underlying genetic substrate [71].

### CMR-based differentiation: Lake Louise 2018 and Padua 2020 criteria

CMR is the central imaging modality for differentiating viral or immune myocarditis from genetic hot-phase episodes, though it is important to recognize that these entities share substantial imaging overlap. Up to 80% of confirmed DSP hot-phase presentations fulfil Lake Louise 2018 criteria for myocarditis [72]. The distinction therefore rests not on whether criteria are met, but on the specific pattern, distribution, and behavior of CMR abnormalities on serial imaging.

Under the Lake Louise 2018 criteria, a T2-based myocardial oedema criterion plus at least one T1-based myocardial injury criterion (elevated native T1 or extracellular volume fraction > 27%, or non-ischemic LGE in ≥ 1 segment) is required; pericardial effusion or LGE serves as a supporting criterion [73]. In viral myocarditis, LGE is typically subepicardial or midfall in the lateral and inferior left ventricle, proportionate to the troponin rise, and regresses on follow-up CMR within 3–6 months. In genetic hot-phase episodes, LGE is frequently extensive relative to the troponin elevation, may adopt a ring-like or epicardial distribution (particularly in DSP and FLNC disease), is biventricular in desmosomal disease involving PKP2 or DSG2, and is stable or progressive on serial imaging [74].

The Padua Task Force criteria (2020) extend the original 2010 Task Force criteria for ACM to incorporate left-dominant and biventricular phenotypes [75]. A critical limitation at the hot-phase stage is that structural criteria are frequently unmet in patients with preserved LVEF and no wall motion abnormality—the typical presentation of DSP-related inflammatory disease. The subepicardial LGE pattern in the left ventricle, particularly in a ring-like, inferior-lateral, or biventricular distribution, is now integral to the diagnosis of left-dominant ACM under the Padua extension, and its presence should prompt genetic evaluation irrespective of whether volumetric or functional criteria are met [76].

CMR red flags warranting genetic evaluation, regardless of Lake Louise status, include: ring-like LV LGE; LGE extent disproportionate to troponin elevation; biventricular or epicardial LGE; persistently elevated extracellular volume fraction (> 27%) on follow-up; LVEF preserved despite extensive fibrosis; and absence of pericardial involvement. The combination of normal LVEF, significant LGE, and ventricular arrhythmia is particularly characteristic of DSP- and FLNC-related disease [77].

### Arrhythmia risk, exercise restriction, and sudden cardiac death

Arrhythmic risk in genetic inflammatory cardiomyopathy is substantially higher than in viral myocarditis and is present across all phases of disease, including the quiescent period between inflammatory episodes. This risk is driven by four overlapping mechanisms: (1) replacement fibrosis creating a fixed re-entry substrate that persists independent of active inflammation; (2) active myocardial oedema during the inflammatory episode lowering the ventricular fibrillation threshold; (3) catecholamine surge during exercise triggering VT in the fibrotic ACM substrate; and (4) mechanical disruption of desmosomal junctions causing Cx43 lateralization and gap junction remodelling [33, 34, 78].

ACM accounts for approximately 10–15% of sudden cardiac death (SCD) in young athletes, and exercise is an independent predictor of disease progression and adverse arrhythmic outcomes in multiple ACM cohorts [79, 80]. This relationship is causal rather than coincidental: high-endurance exercise accelerates desmosomal protein shedding, promotes fibroadipose replacement, and increases arrhythmic burden in genotype-positive individuals [46].

During the acute hot phase, complete restriction from all exercise—including recreational activity—is mandatory until troponin has normalized and CMR T2 signal has resolved, with a minimum duration of three months. In genotype-positive patients in the quiescent phase, competitive sport is contraindicated. Return to moderate-intensity recreational activity may be considered after at least three to six months of documented quiescence, provided LVEF ≥ 50% and RVEF ≥ 45%, no sustained VT or NSVT burden > 500 VPBs/24 h on Holter monitoring, absence of exercise-provoked VT on formal exercise testing, and stable or reduced LGE extent on repeat CMR [81, 82]. These criteria are substantially more stringent than those applied to viral myocarditis. Prolonged ambulatory monitoring—including insertable cardiac monitors—should be considered in high-risk genotype-positive patients given the disproportionately high ventricular ectopic burden that characterizes these patients compared to viral myocarditis.

High-risk features warranting ICD discussion include: unexplained syncope; aborted sudden cardiac arrest or documented VF; sustained VT; LVEF < 35% or RVEF < 35%; and extensive LGE (> 20% LV mass) with documented arrhythmia. In *DSP* and *LMNA* variant carriers, the threshold for ICD implantation is generally lower, as both genotypes are associated with disproportionately high arrhythmic risk relative to structural disease; ICD implantation may be appropriate even with preserved LVEF if significant LGE and NSVT are present [83, 84]. All ICD decisions should involve shared decision-making with the patient and multidisciplinary input from an inherited cardiac disease team.

### Therapeutic management

Acute episode management follows standard myocarditis protocols: NSAIDs and colchicine for the pericarditic component, activity restriction, and guideline-directed heart failure therapy if LVEF is reduced [85]. The management of genotype-positive inflammatory cardiomyopathy diverges from standard myocarditis care in several respects.

Immunosuppression with corticosteroids or steroid-sparing agents is not recommended routinely in genotype-positive patients, as no randomized trial evidence supports this approach in desmosomal inflammatory cardiomyopathy, and corticosteroids carry theoretical risks of accelerating fibrofatty replacement [86]. This represents a fundamental therapeutic dilemma: if the patient presents with active inflammation and a pathogenic *DSP* variant, the clinician faces the risk of withholding immunosuppression that might be beneficial in primary autoimmune myocarditis, versus initiating therapy that may worsen the ACM substrate. Current consensus favors avoiding corticosteroids unless CS or autoimmune myocarditis has been confirmed by EMB and viral PCR negativity. Mechanism-targeted approaches—including IL-1 and IL-6 pathway inhibition—are under active investigation but should not be applied outside clinical trials, given the absence of human evidence of benefit in genotype-positive inflammatory cardiomyopathy [87].

Beta-blockade is indicated in genotype-positive patients regardless of LVEF, given its anti-arrhythmic benefit in the ACM substrate. ACEi or ARB therapy should be initiated if LVEF falls below 50%, but current evidence does not support altering guideline-directed therapy based on genotype alone in the absence of established ACM structural criteria.

### Family screening and long-term surveillance

Family screening is mandatory once a pathogenic or likely pathogenic variant is identified in a proband. All first-degree relatives should undergo cascade genetic testing. Gene-positive relatives require baseline clinical evaluation including CMR, 12-lead ECG, and Holter monitoring, with annual surveillance thereafter, even in the absence of symptoms [65].

Patients with confirmed or suspected genetic inflammatory cardiomyopathy require lifelong specialist follow-up at an inherited cardiac disease centre. The recommended surveillance schedule includes clinical review and ECG every six months; 48-h Holter monitoring and symptom-limited exercise testing every six months in the first two years and annually thereafter; and CMR with T1/T2 mapping and LGE assessment annually. Any new episode of troponin elevation, arrhythmia, or symptoms should trigger urgent re-evaluation with repeat CMR and re-stratification of SCD risk.

### Limitations of the current evidence and of this review

Several important limitations must be acknowledged. First, this review is narrative rather than systematic, and the conclusions are vulnerable to selection bias inherent in the choice of included literature. Second, the overwhelming majority of the evidence base consists of retrospective observational cohorts and tertiary referral registries with inherent case-mix bias, where variant prevalence is substantially higher than in unselected community populations. Third, causality between genetic variants and inflammatory episodes has not been established in prospective human studies; the mechanistic models described—particularly the autoimmune cascade—represent working hypotheses supported by experimental data but not proven in human cardiac tissue. Fourth, clinically meaningful outcome data from long-term perspective follow-up of genotype-positive patients after myocarditis episodes are absent. Fifth, the field lacks standardized, validated assays for anti-DSG2 and related autoantibodies suitable for routine clinical use. These limitations collectively imply that the paradigm shift described in this review remains evidence-informed but not yet evidence-proven, and that the recommendations offered represent expert opinion informed by best available data rather than guideline-level evidence.

## Conclusions

The intersection between myocarditis and inherited cardiomyopathies challenges the view that myocardial inflammation is invariably an isolated acquired disorder. Evidence drawn predominantly from retrospective registries and tertiary referral cohorts indicates that, in a subset of patients, myocarditis-like presentations may represent an early manifestation of underlying desmosomal ACM. The strength of support for the key propositions within this paradigm, however, differs and warrants explicit differentiation.

Two observations are now reasonably supported by available data. First, desmosomal gene variants are enriched among patients presenting with myocarditis—particularly those with recurrent inflammatory episodes, ventricular arrhythmias disproportionate to myocardial inflammation, subepicardial or ring-like LGE on CMR, or a family history of ACM or sudden cardiac death. The magnitude of this enrichment varies substantially by case selection: from 8% in population-based unselected cohorts to 22–45% in referral and complicated-myocarditis populations. Second, longitudinal data suggest that recurrent inflammatory episodes may, in some patients, precede the structural phenotype of ACM by several years, with cycles of inflammatory injury potentially contributing to replacement fibrosis and arrhythmogenic remodeling. Whether this trajectory is inevitable, genotype-specific, or modifiable remains undefined.

A third proposition—that autoimmune responses to desmosomal epitopes directly drive disease progression—is supported by experimental data from preclinical models but lacks direct proof of pathogenicity in human cardiac tissue. Anti-DSG2 antibodies and related autoantibodies should not currently be used as diagnostic or therapeutic targets outside research settings, and their clinical significance requires prospective evaluation.

These evidential distinctions translate into calibrated clinical guidance. Targeted genetic testing is supported by available evidence in patients with myocarditis who have one or more high-probability features (recurrence, ventricular arrhythmia, subepicardial or biventricular LGE, family history). In these patients, a cardiomyopathy gene panel and ACM-focused workup—including signal-averaged ECG, ambulatory monitoring, and first-degree family screening—is clinically reasonable, though prospective data demonstrating impact on hard outcomes are not yet available. EMB retains a role when GCM, CS, or eosinophilic myocarditis must be excluded. Current evidence does not support modifying guideline-directed therapy based on genotype alone before overt ACM criteria are met.

Recognizing that recurrent inflammatory episodes may be the first expression of ACM—and responding with targeted genetic evaluation, arrhythmic surveillance, and family screening—is an evidence-informed and clinically actionable step. Asserting that autoimmune mechanisms are established drivers of progression, or that mechanism-based therapies are ready for clinical use, is not warranted by current evidence and should await prospective validation.

**Fig. 2:**
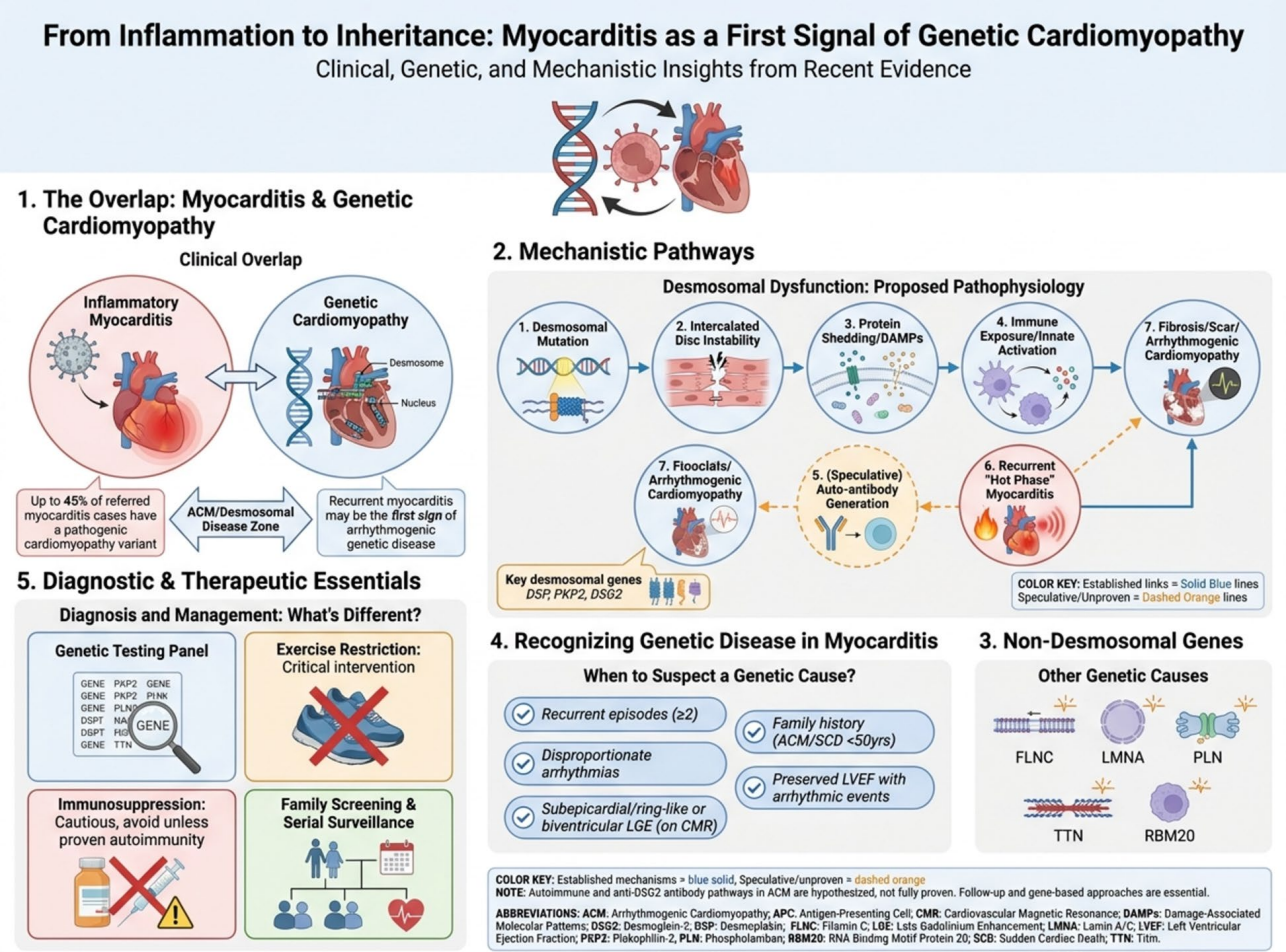
This figure presents the results obtained from the experiments, summarizing evidence in this article

Figure 2 summarizes the key facts in the manuscript.

## Data Availability

No datasets were generated or analysed during the current study.

## Abbreviations

ACM: Arrhythmogenic cardiomyopathy
ARVC: Arrhythmogenic right ventricular cardiomyopathy
CMR: Cardiac magnetic resonance
Cx43: Connexin-43
DAMPs: Damage-associated molecular patterns
DCM: Dilated cardiomyopathy
DSC2: Desmocollin-2
DSG2: Desmoglein-2
DSP: Desmoplakin
ECG: Electrocardiogram
ECV: Extracellular volume fraction
EMB: Endomyocardial biopsy
FLNC: Filamin C
GCM: Giant cell myocarditis
ICD: Implantable cardioverter-defibrillator
ILR: Implantable loop recorder
LGE: Late gadolinium enhancement
LMNA: Lamin A/C
LV: Left Ventricle
LVEF: Left ventricular ejection fraction
NF-κB: Nuclear factor kappa-B
NSVT: Non-sustained ventricular tachycardia
PKP2: Plakophilin-2
PLN: Phospholamban
RV: Right ventricle
RVEF: Right ventricular ejection fraction
SCA: Sudden cardiac arrest
SCD: Sudden cardiac death
TF: Task force
TLR: Toll-like receptor
TTN: Titin
VF: Ventricular fibrillation
VPB: Ventricular premature beat
VT: Ventricular tachycardia
VUS: Variant of uncertain significance
